# Rectal Ulcer After Rectal Washing With a Bidet Toilet

**DOI:** 10.14309/crj.0000000000000754

**Published:** 2022-02-15

**Authors:** Jeongmin Choi

**Affiliations:** 1 Department of Internal Medicine, Division of Gastroenterology, Sanggye Paik Hospital, Inje University College of Medicine, Nowon-gu, Seoul, Korea

## CASE REPORT

Bidets are popular in some countries of Asia-Pacific and Europe. Because of the importance of bathroom hygiene and proper cleaning of soiled areas after bowel movement, the demand for bidets has increased. However, inappropriate use of bidets can damage the colonic mucosa. Here, we report a case of rectal mucosal damage due to inappropriate bowel cleansing habits using a bidet.

A 59-year-old man came to our hospital with rectal ulcerations on screening colonoscopy. He had no significant medical history. He did not complain of fever, abdominal pain, hematochezia, constipation, diarrhea, or pain during defecation. He had soft bowel movements every day without straining. He underwent a screening colonoscopy.

**Figure 1.**
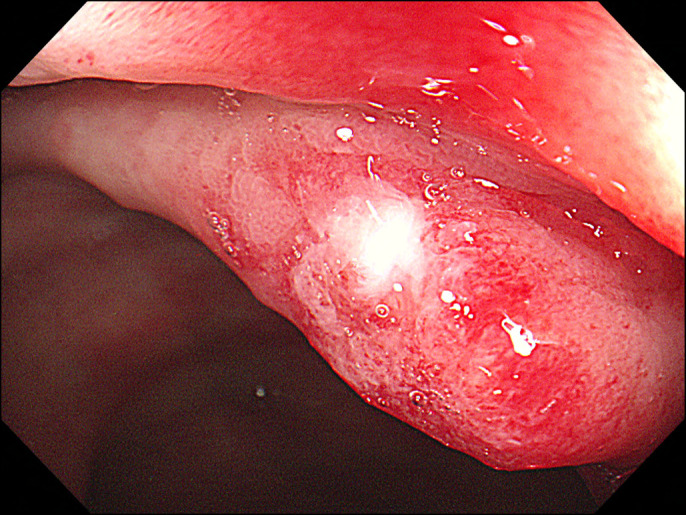
Ulceration surrounded by a hyperemic edematous mucosa of the rectum near the anus on colonoscopy.

Colonoscopy revealed several ulcers that were surrounded by hyperemic and edematous mucosa of the rectum near the anus (Figure 1). Possible endoscopic differential diagnoses included solitary rectal ulcer syndrome, ischemic colitis, malignancy, and infectious protocolitis, such as amebic colitis.^1,2^ Biopsy revealed an ulceration and regenerating atypia with hyperplastic epithelium. However, no definitive diagnosis was made.

**Figure 2.**
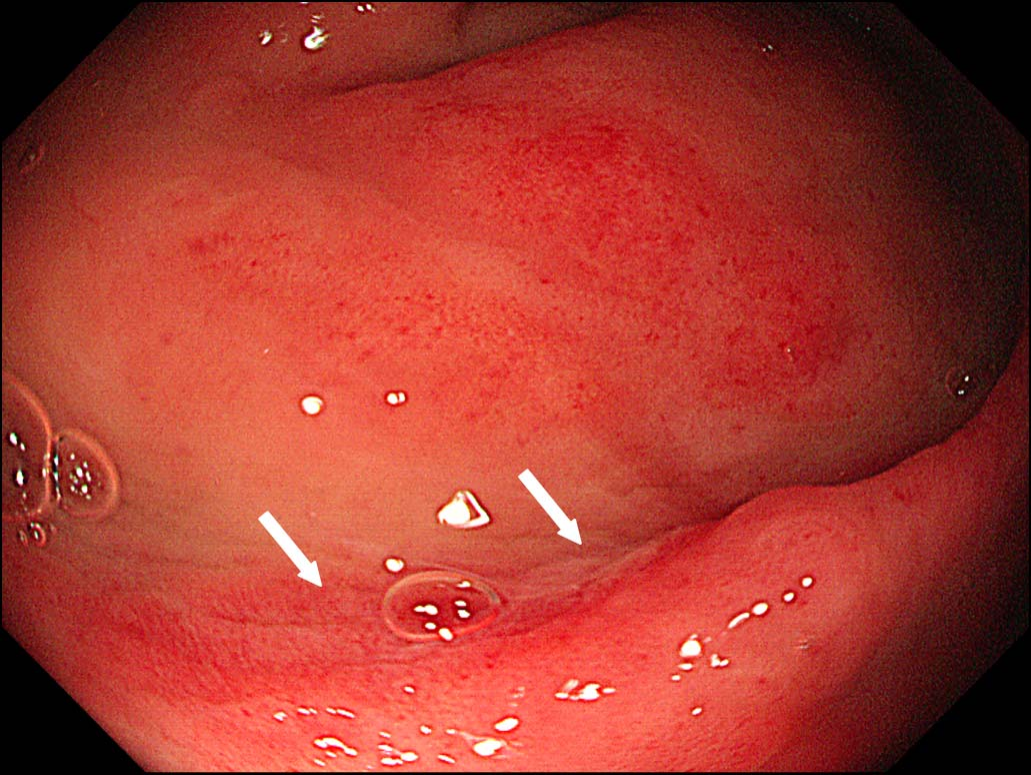
A healing stage of rectal ulceration at 3-month follow-up sigmoidoscopy (arrows).

**Figure 3.**
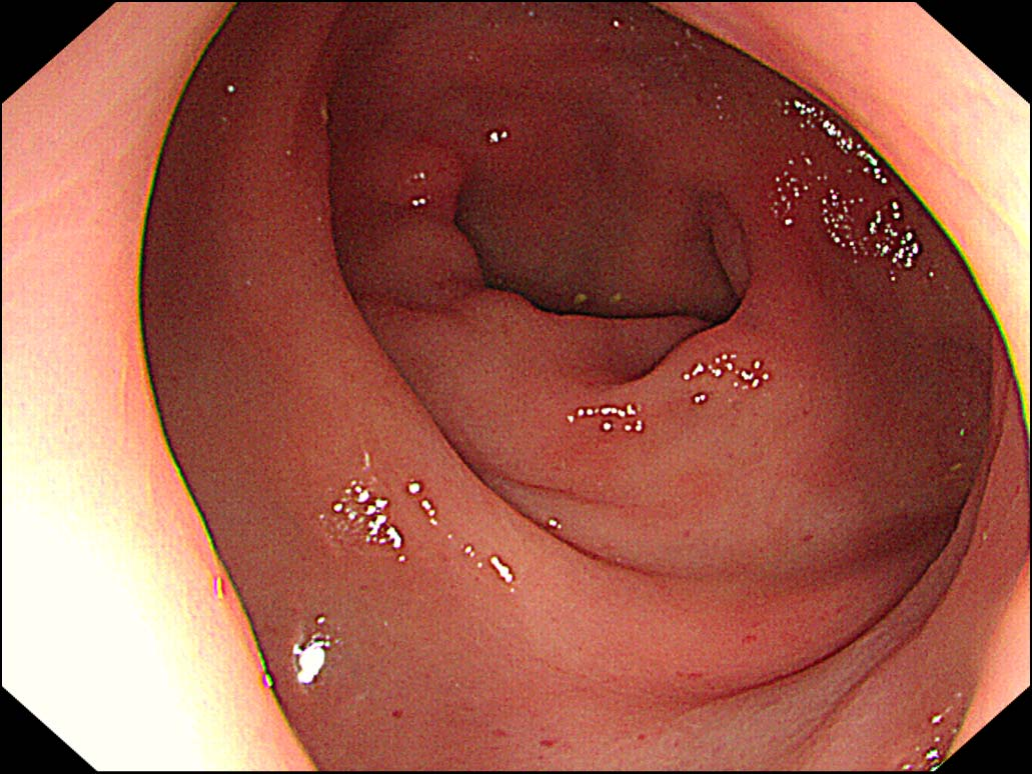
At the 2-year follow-up, no abnormal lesions were found on sigmoidoscopy.

Further history taking revealed that the patient used an electric bidet toilet with controllable water temperature, water jet pressure, and nozzle movement. After bowel movement, he had a habit of opening his anus to clean the rectum with the highest water pressure for 1 year. He was advised to stop using the bidet for rectal cleansing purpose and to clean the anus with a medium water pressure. The physician found a significant improvement in the rectal ulceration at 3-month follow-up sigmoidoscopy (Figure 2). At the 2-year follow-up, no abnormal lesions were found on sigmoidoscopy (Figure 3).

Despite the widespread use of bidets, few studies have investigated the effect of bidet use on anorectal disease. A case of rectal mucosal prolapse syndrome due to bidet overuse has been reported previously.^3^ There was an increased incidence of hemorrhoids and pruritus ani (intense itching affecting the anorectal area) associated with habitual users of bidets.^4^ However, the causal relationship was unknown because the data were based on a survey.^4^ Theoretically, strong water pressure can strip the oil that protects the anal skin and damage the anorectal area. The anorectal area becomes rough, dry, damaged, and susceptible to infection. It is not known how high water jet pressure can damage the anorectal mucosa.

Some manufactures advocate high water jet pressure can aid defecation. However, a study on anorectal pressures during bidet use showed that high-pressure jets can cause reflex contraction of the anal sphincter, which can lead to dyssynergic defecation.^5^ The patient unintentionally experienced rectal ulcers due to overzealous bidet cleaning of the rectal area. Physicians should educate the patients that washing the rectum with the strongest bidet water pressure can damage the anorectum.

## DISCLOSURES

Author contributions: J. Choi is the sole author of this article and is the article guarantor.

Financial disclosure: None to report.

Informed consent was obtained for this case report.
